# Speech Analysis by Natural Language Processing Techniques: A Possible Tool for Very Early Detection of Cognitive Decline?

**DOI:** 10.3389/fnagi.2018.00369

**Published:** 2018-11-13

**Authors:** Daniela Beltrami, Gloria Gagliardi, Rema Rossini Favretti, Enrico Ghidoni, Fabio Tamburini, Laura Calzà

**Affiliations:** ^1^Interdepartmental Centre for Industrial Research in Health Sciences and Technologies, University of Bologna, Bologna, Italy; ^2^Clinical Neuropsychology Unit, Arcispedale S. Maria Nuova di Reggio Emilia, Reggio Emilia, Italy; ^3^Department of Classical Philology and Italian Studies, University of Bologna, Bologna, Italy; ^4^Department of Pharmacy and Biotechnology, University of Bologna, Bologna, Italy

**Keywords:** cognitive decline, language, Natural Language Processing, preclinical Alzheimer, speech analysis, mild cognitive impairment

## Abstract

**Background:** The discovery of early, non-invasive biomarkers for the identification of “preclinical” or “pre-symptomatic” Alzheimer's disease and other dementias is a key issue in the field, especially for research purposes, the design of preventive clinical trials, and drafting population-based health care policies. Complex behaviors are natural candidates for this. In particular, recent studies have suggested that speech alterations might be one of the earliest signs of cognitive decline, frequently noticeable years before other cognitive deficits become apparent. Traditional neuropsychological language tests provide ambiguous results in this context. In contrast, the analysis of spoken language productions by Natural Language Processing (NLP) techniques can pinpoint language modifications in potential patients. This interdisciplinary study aimed at using NLP to identify early linguistic signs of cognitive decline in a population of elderly individuals.

**Methods:** We enrolled 96 participants (age range 50–75): 48 healthy controls (CG) and 48 cognitively impaired participants: 16 participants with single domain amnestic Mild Cognitive Impairment (aMCI), 16 with multiple domain MCI (mdMCI) and 16 with early Dementia (eD). Each subject underwent a brief neuropsychological screening composed by MMSE, MoCA, GPCog, CDT, and verbal fluency (phonemic and semantic). The spontaneous speech during three tasks (describing a complex picture, a typical working day and recalling a last remembered dream) was then recorded, transcribed and annotated at various linguistic levels. A multidimensional parameter computation was performed by a quantitative analysis of spoken texts, computing rhythmic, acoustic, lexical, morpho-syntactic, and syntactic features.

**Results:** Neuropsychological tests showed significant differences between controls and mdMCI, and between controls and eD participants; GPCog, MoCA, PF, and SF also discriminated between controls and aMCI. In the linguistic experiments, a number of features regarding lexical, acoustic and syntactic aspects were significant in differentiating between mdMCI, eD, and CG (non-parametric statistical analysis). Some features, mainly in the acoustic domain also discriminated between CG and aMCI.

**Conclusions:** Linguistic features of spontaneous speech transcribed and analyzed by NLP techniques show significant differences between controls and pathological states (not only eD but also MCI) and seems to be a promising approach for the identification of preclinical stages of dementia. Long duration follow-up studies are needed to confirm this assumption.

## Introduction

The increasing prevalence of dementia among the elderly population is a major societal challenge, leading to a growing demand of diagnostic services for defects in memory and cognitive performance. One major diagnostic focus would be the early distinction among memory and cognitive complains likely to evolve as neurodegenerative disease, from functional symptoms, or non-neurological disorders. In this area, terminology and diagnostic criteria are still under discussion. For example, the general description of “a person reporting the feeling of an impairment of the cognitive function” is named “subjective cognitive impairment” or “subjective cognitive decline,” or “subjective memory complains,” or “functional memory disorder” etc., and robust diagnostic criteria are not yet available (Stewart, 2012; Burmester et al., 2016), although a specific working group proposed research criteria (Jessen et al., 2014). Moreover, extensive research over the past decades in the dementia field, have recognized “an intermediate state of cognitive function between the changes seen in aging and those fulfilling the criteria for dementia and often Alzheimer disease (AD),” named Mild Cognitive Impairment (MCI, Petersen, 2011). From a clinical point of view, MCI has been then categorized in two major subtypes, i.e., amnestic MCI (aMCI) and non-amnesic MCI (naMCI), each of them including one (single) or more (multiple) cognitive domains (Petersen et al., 2014), which might or not evolve in dementia. When evolving as dementia, MCI is preceded by a very long biological history of the disease, as suggested by longitudinal models of the alteration of AD biomarkers including Ab42 and tau in the cerebrospinal fluid (CSF), amyloid deposition at PET, MRI alterations and FDG PET abnormalities (Selkoe and Hardy, 2016). This leads to the identification of new entities to be considered as research criteria, referred as “prodromal AD” by the International Working Group-2 (IWG-2; Dubois et al., 2014) and “MCI due to AD” by the AD group at the National Institute of Aging-Alzheimer Association (NIA-AA; Albert et al., 2011). This preclinical period could offer a window of opportunity for drug development, risk assessment, and prevention (Calzà et al., 2015; Epelbaum et al., 2017; Ritchie et al., 2017).

Overall these studies addressed research attention on the feasibility of detecting early cognitive changes, and several initiatives and researches are in progress, focusing on identifying the best predictive among the available cognitive tests (Mortamais et al., 2017). Memory is probably the most investigated domain. Episodic memory functioning seems to be a robust predictor of dementia in prospective studies based on *in vivo* amyloid imaging (Bäckman et al., 2005; Hedden et al., 2013). Some aspects of language have also been the subject of growing interest, and most of these studies focused on verbal ability, verbal learning and memory, naming, category or letter verbal fluency, verbal episodic memory, etc. The evaluation of the linguistic functions is usually performed by means of traditional pencil-and-paper or corresponding computer-assisted tests (Ostberg et al., 2005; Duong et al., 2006; Cuetos et al., 2009; Joubert et al., 2010; Pakhomov et al., 2012). Composite scores exploring both memory and language have also been proposed, such as the Alzheimer's Disease Cooperative Study Preclinical Alzheimer Cognitive Composite (ADCS-PACC) (Donohue et al., 2014) and the Alzheimer prevention initiative (API) composite score (Langbaum et al., 2014). The API score is composed of seven test scores, i.e., category fluency—fruits and vegetables—, Boston naming test, Logical Memory-delayed recall, east Boston naming test immediate recall, Ravens progressive matrices subset, symbol digit modalities, and the Mini-Mental State Examination (MMSE) orientation to time items. Composite scores are now being used as primary end-point in secondary prevention trials in AD involving presenilin 1 E280A mutation carriers (API trial, Ayutyanont et al., 2014) or in anti-amyloid treatments in asymptomatic individuals that show early amyloid accumulation (Donohue et al., 2014).

While sometimes significant differences between the MCI and normal elderly participants have been recognized by these tests, the range of variation of the scores in MCI often overlaps with that of normal people, making their clinical use unreliable in categorizing individual participants (Taler and Phillips, 2008). Even more confused results emerged from the few studies on Subjective Cognitive Complaints (Martins et al., 2012).

New perspectives are being opened up by the interest toward computerized analysis of spoken language (Natural Language Processing techniques), together with the availability of numerous algorithms for analysis and classification of “speech.” The experience gained in the “Electronic linguistic corpora” studies, chosen by virtue of their representativeness in characterizing a particular language or linguistic variety, are now opening up new perspectives for language analysis in clinical contexts, also considering that these approaches might quantify many aspects of language, both at the segmental and suprasegmental level, such as prosody and rhythm, that are not explored by conventional language tests.

When applied to “pathological language” (i.e., linguistic productions of subjects affected by a developmental or acquired speech and language disorder), this approach and related technologies would also have the significant advantage of representing a natural and spontaneous language record, outside the diagnostic set-up of the conventional neuropsychological test of language, potentially applicable to large sections of the population using low-cost tools.

With this connection established, we intended to investigate whether the analysis of the spontaneous speech performed by Natural Language Processing techniques could reveal alterations of the language performance in early cognitive decline. This proof-of-concept study analyzed, by using the Natural Language Processing techniques, the spontaneous speech used by the participants to answer to three specific tasks, i.e., the description of a drawing, details of a last dream and the description of a working day. The study included 96 participants, divided into a control group (CG, *N* = 48) and three pathological groups (PG), e.g., amnestic MCI (aMCI, *N* = 16), multiple domain MCI (mdMCI, *N* = 16) and early dementia (eD, *N* = 16).

## Materials and methods

### Participants

The study was approved by the Ethical Committee of Azienda Ospedaliera Reggio Emilia (n. 2013/0013438). We enrolled 96 participants (48 males; 48 females) between the ages of 50 and 75 (according to the age range of people admitted to our outpatient clinical service) and with at least a junior high school certificate (8 years of education) or primary school certificate (5 years of education) with high intellectual interests throughout the life span. All of them provided informed and written consent. The sample was composed of a Control Group (CG) and a Pathological Group (PG). The CG included 48 participants. The PG included 48 participants from two outpatient clinical services involved in care and diagnostic evaluation of cognitive disorders and dementia. Inclusion criteria are outlined in Table 1.

**Table 1:** Inclusion criteria for participant enrollment in control and pathological groups.

**Control group (CG)**	**Pathological groups (PG)**
- MMSE Raw Score (RS) ≥ 24; - MoCA RS ≥ 18; - No current or previous neurological pathologies; - No blindness, deafness or other serious sensory impairment; - No intellectual disability; - Adequate verbal comprehension; - No attentional or perceptual impairments; - No current or previous history of serious psychiatric diseases; - No familiarity for early onset dementia (onset before age 65).	- MMSE ≥ 18; - History of Mild Cognitive Impairment or early Dementia; - No blindness, deafness or other serious sensory impairment; - No intellectual disability; - Adequate verbal comprehension (in order to understand the instructions); - No current or previous history of serious psychiatric diseases. **Additional criteria for aMCI subgroup:** - At least one long-term memory test impaired (ES 0 or under cut off for age and education) - No problem in activities of daily living Additional criteria for mdMCI subgroup: - Two or more cognitive areas affected (ES 0 or under cut off for age and education in the tests regarding these areas) - No problem in activities of daily living **Additional criteria for eD subgroups:** - Two or more cognitive areas affected - Need of support for one or more activities of daily living

The PG refers to three categories: (i) amnestic Mild Cognitive Impairment (aMCI; 16 participants: 8 females, 8 males) characterized by an isolated memory deficit detected by standardized tests; (ii) multiple-domain MCI (mdMCI; 16 participants: 8 females, 8 males) where two or more cognitive abilities are affected (12 amnestic-md-MCI; 4 non-amnestic-md-MCI); (iii) early Dementia (e-D; 16 participants: 8 females, 8 males). Within this group, four sub-categories can be identified: probable Alzheimer Dementia (AD; six cases); Fronto-Temporal Dementia (FTD; one case of Primary Progressive Aphasia—PPA; one subject presenting the executive and behavioral variant; one case of semantic dementia); Mixed Dementia (MD; six cases) and Lewy Body Dementia (LBD; one case).

In the first two conditions (MCI), the cognitive changes are serious enough to be detected by neuropsychological assessment, but not so severe to interfere with everyday activities, as evaluated by the conventional scales (e.g., ADL -Activities of Daily Living-Petersen, 2004; Winblad et al., 2004); in the eD, the cognitive deficits partially interfere in everyday life (however, the Mini Mental State Examination score is equal or greater than 18).

### Conventional neuropsychological evaluation

All the participants of the CG and PG were requested to complete the anamnestic interview (anagraphic data; occupation/retirement; children; familiarity with neurodegenerative pathologies; clinical history and pharmacotherapy), a neurological assessment and other medical examinations planned in the diagnostic work-up and the traditional cognitive battery aimed to evaluate several cognitive domains: logic, memory, attention, language, visuo-spatial, praxic, and executive functions.

The battery was composed of those tests which are most used in the clinical practice to assess cognitive decline (Velayudhan et al., 2014; Tsoi et al., 2015), with an Italian standardization and short administration time. In particular: Mini Mental State Examination (MMSE); Phonemic (PF) and Semantic (SF) verbal fluency tests; Clock Drawing Test (CDT), Montreal Cognitive Assessment (MoCA); General Practitioner Assessment of Cognition (GPCog). MMSE is a 30-point questionnaire providing measures of orientation, encoding (immediate memory), short-term memory as well as language functioning; Phonemic and Semantic Fluency test are commonly used verbal tasks in which the subject is requested to produce, in 1 min, words relevant to a given category; they allow a quick evaluation of the lexical access, whose functioning can be compromised early on in neurodegenerative disorders (Auriacombe et al., 2006; Clark et al., 2009). The verbal fluency test is included in the ACE-R (Mioshi et al., 2006), which is indicated as an accurate tool to identify MCI and early dementia. CDT is a brief cognitive task which mainly assesses praxis and planning abilities. It is very accurate in detecting dementia, whilst remains unclear in its accuracy to recognize MCI (Lee et al., 2011); MoCA is a 30-point test which assesses several cognitive domains such a verbal memory, visuospatial and executive abilities, attention and language; GP-Cog is a brief screening tool for cognitive impairment, designed for general practitioners and primary care physicians. It is composed of two different parts: the patient assessment (registration and recall of verbal information, temporal orientation, visuospatial abilities, and language); a caregiver interview which has to be submitted only when the scoring of the first one (range 0–9) is lower than 9. For this reason, in this study we considered and reported only the first part.

In order to compare the cognitive performances of the different groups (CG vs. aMCI vs. mdMCI vs. eD), we corrected the raw scores of the different cognitive tests (MMSE, MoCA, PF, SF) for age and education, as indicated in the respective standardization procedure (SF, Novelli et al., 1986; MMSE, Measso et al., 1993; PF, Carlesimo et al., 1995; MoCA, Conti et al., 2015), thus obtaining a standardized score, as it is usually done in clinical practice. For those (CDT, GP-Cog), which lack correction, we used the original raw scores. For each test the raw scores were transformed into adjusted scores (with correction for age and education) and classified as Equivalent Scores (ES), a 5-point scale that offers a solution to the problem of standardizing neuropsychological scores after adjustment for age and education. ES = 0 reflects a pathological performance, ES > 0 (1–4) means performance in the range of normality. Subjects with ES = 0 in the single memory domain or in more different cognitive areas, autonomous in the activities of daily living, MMSE score ≥18, were respectively classified as aMCI or mdMCI. Subjects with ES = 0 in more than one test, with need of support in one or more activities of daily living, MMSE score ≥18, were classified as e-D. Subjects with MMSE < 18 were discarded.

### Speech analysis

All participants were required to record their spontaneous speech during the execution of three tasks, elicited by these input sentences: “Could you please describe this picture?” (the picture illustrated a living room with some characters carrying out certain actions; Ciurli et al., 1996); “Could you please describe a typical working day?”; “Could you please describe the last dream you remember?” Spontaneous speech samples were recorded in a quiet room with an Olympus—Linear PCM Recorder LS-5 (in WAV files; 44.1 KHz, 16 bit) placed on a table in front of the subject.

Speech samples were collected during test sessions in the form of audio files and were manually transcribed in order to produce the dialogue orthographic transcription through the use of the *Transcriber* software package (http://trans.sourceforge.net). We chose the “utterance” as the reference unit in the speech continuum, defined as the counterpart of a speech act (Finegan, 2011). The latter can be considered as “*the minimal linguistic entity that can be pragmatically interpreted*.” Utterances are demarcated by prosody in the speech flow, therefore the identification of their boundaries is achieved through the detection of “prosodic breaks.” As a matter of fact, a large body of evidence suggests that the perception of prosodic breaks is a function of the simultaneous activation of some acoustic cues (e.g., F0 reset, final lengthening, drop in intensity, pause, initial rush in the next prosodic unit) reaching high inter-rater agreement in annotation. The manual detection of such breaks has been achieved by listening the utterances and splitting the entire dialogues into turns. In addition, during the transcription process, a series of paralinguistic phenomena such as empty and filled pauses (e.g., “mmh,” “eeh,” “ehm”), disfluencies (e.g., hesitation, stuttering, false start, lapsus…) and non-verbal phenomena (e.g., inspirations, laughs, coughing fits, throat clearing) were annotated inserting also temporal information. In order to consistently transcribe subjects' productions, we developed strict transcription guidelines imposing the transcribers to comply to the designed transcription protocol. This ensured consistency among them during the entire project.

The word turns of the test subjects were isolated, removing all speech fragments containing speech produced by the interviewer, and the selected utterances underwent automatic morphological and syntactic annotation. More precisely, they were Part-of-Speech tagged and syntactically parsed with the dependency model used by the Turin University Linguistic Environment—TULE (Lesmo, 2007), based on the TUT–Turin University TreeBank tagset (Bosco et al., 2000) in order to explicit all the morphological, syntactic and lexical information. Afterwards, all the automatically inserted annotations were manually checked to remove all the errors introduced by the annotation procedures.

A multidimensional parameter computation was performed on the data, evaluating rhythmic, acoustic, lexical, and morpho-syntactic quantitative features of the spoken utterances.

The Table in Supplementary Material outlines the complete list of the linguistic/stylometric cues considered in the study: indexes proposed in the literature as statistically relevant on languages other than Italian. Moreover, some new parameters have been tested. More precisely, we checked in the cited literature for linguistic features that, in the referred study, were able to successfully discriminate in a statistically significant way between controls and MCI or eD subjects. All these linguistic features have been considered for this study and proper computational tools for computing them have been developed and carefully tested.

With regard to the parameters derived from the speech acoustics, we used the “ssvad” Voice Activity Detector (Mak and Yu, 2014), especially developed for interview speech, to segment the recordings and identify speech vs. non-speech regions. Moreover, we used a forced alignment system developed for this study by using the Kaldi-DNN-ASR (http://kaldi.sourceforge.net/about.html) package trained on the APASCI Italian Corpus (Angelini et al., 1994), for obtaining the temporally aligned phonetic transcriptions needed to compute various rhythmic and acoustic features.

### Data availability and statistical analysis

All data are stored in anonymous form at the experimentation sites. Due to the Italian privacy policy, data (recordings, transcriptions, anagraphic, and clinical data, etc.) are protected and availability restricted to the project participants. Demographic variables are presented in Table 2, as mean ± SD Results of the neuropsychological tests are presented as median and interquartile range. Because of the small sample size, the non-parametric Mann-Whitney *U*-test was used to compare performances in neuropsychological tests and language between two groups of subjects (eD vs. CG), and the non-parametric Kruskal-Wallis test with Dunn's multiple comparison having CG as control, when significant, was used to compare aMCI and mdMCI vs. CG. A probability level of *P* < 0.05 was considered to be statistically significant. All statistical tests were two-sided. The Prism version 6.0 (GraphPad, La Jolla, CA) was used for the statistical analysis.

**Table 2:** Level of education and demographic characteristics of participants.

			**Age**	**Years of education**
		***n***	**Mean ± SD**	**Mean ± SD**
Control group	CG	48	61.60 ± 6.93	13.00 ± 3.92
Pathological group	a-MCI	16	64.19 ± 7.44	11.00 ± 4.00
	md-MCI	16	64.50 ± 7.47	11.56 ± 4.79
	e-D	16	66.38 ± 6.70	9.38 ± 4.01*

The statistical analysis was performed by comparing CG vs. aMCI, mdMCI, and eD by the non-parametric Kruskal-Wallis tests; ^*^p < 0.05. aMCI, amnesic mild cognitive impairment; CG: control group; eD, early dementia; mdMCI, multiple domain mild cognitive impairment.

## Results

### Participants and neuropsychological tests

The focus of the study was the analysis of spontaneous speech in MCI subgroups, compared to healthy CG. We included aMCI and mdMCI, being the memory domain the isolated defect in aMCI, while other cognitive domains are affected in mdMCI. This would identify the best candidate to reveal subclinical disorders in speech production analysis. The eD group was introduced as frankly pathological control.

Age and level of education of enrolled participants in the CG and the PG (aMCI, mdMCI, eD) are reported in Table 2. The statistical analysis (the non-parametric Kruskal-Wallis tests with Dunn's multiple comparison) indicated that no age differences were observed between the subgroups, while the level of education of the eD group is significantly lower compared with the CG (*p*-value: 0.0171).

**Figure 1:**
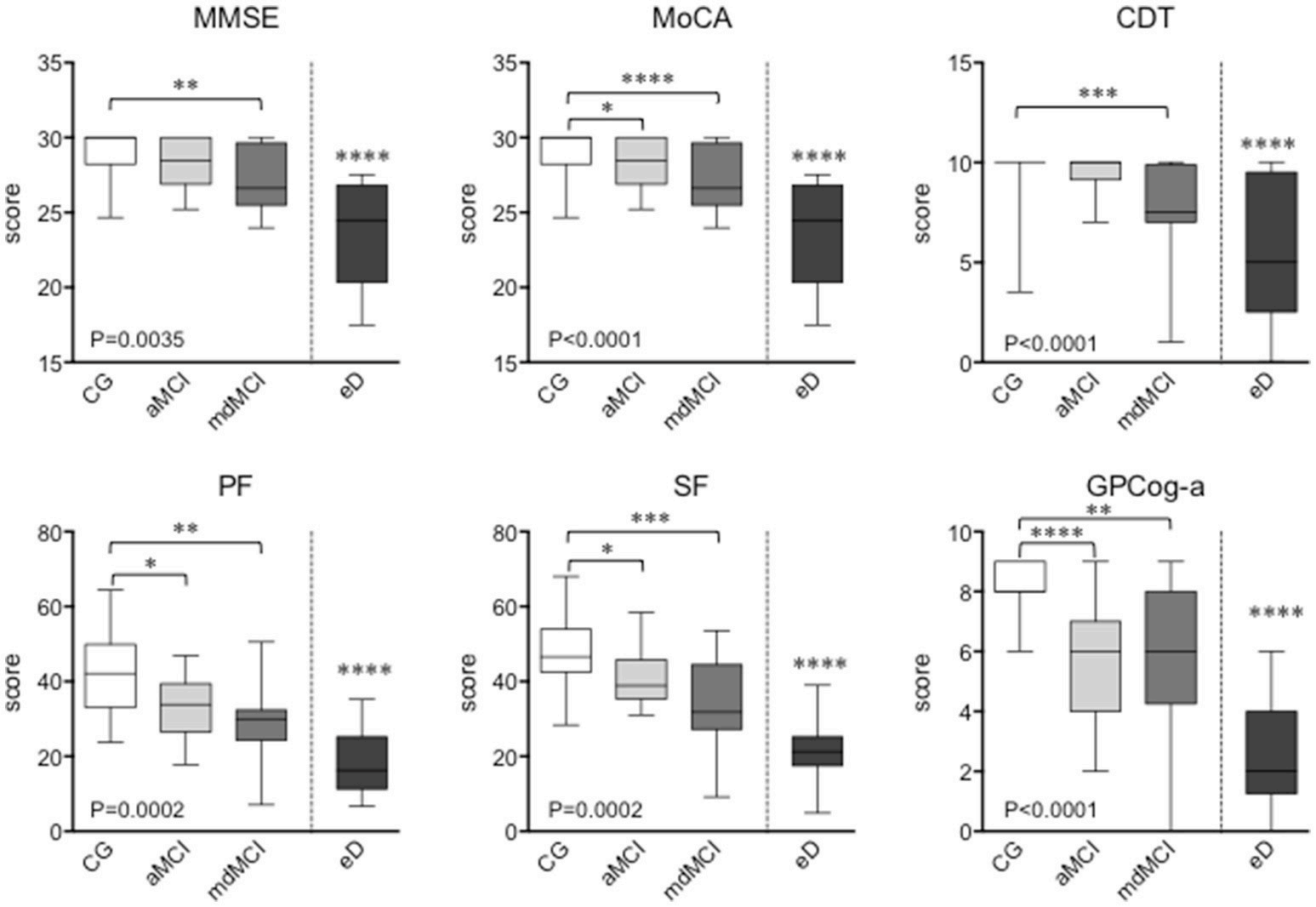
Results of the conventional neuropsycological test performed at the enrolment of the study. The graphs report median and interquartile range. The statistical analysis was performed by the non-parametric Kruskal-Wallis test with Dunn's multiple comparison having CG as control, where *p < 0.05; **p < 0.01; ***p < 0.001; ****p < 0.0001. MMSE, Mini Mental State Examination; MoCA, Montreal Cognitive Assessment; CDT, Clock Drawing Test; GPCog-a, General Practitioner Assessment of Cognition; PF, Phonemic verbal fluency tests; SF, Semantic verbal fluency tests.

Results of the conventional neuropsychological examination are summarized in Figure 1. MCI subgroups and eD group were separately compared to the CG, by the Kruskal-Wallis tests followed by multiple comparison, and Mann-Whitney *U*-test, respectively. As expected, the mean scores of the CG result significantly higher than those obtained by the eD in all tests. GP-Cog is the best test that can discriminate between controls and aMCI. This result is probably due to the fact that aMCI is defined as an isolated memory deficit, and GP-Cog is mainly based on memory performance. MoCA, PF, and SF recognize both aMCI and mdMCI, even with a substantial greater statistical power in mdMCI. MMSE and CDT can discriminate between CG and mdMCI, but they cannot discriminate CG from aMCI.

### Spontaneous speech analysis

The natural language in CG and PG was explored by three different tasks, e.g., two narrative tasks elicited by specific questions (“Could you please describe a typical working day?” “Could you please describe the last dream you remember?”), and a descriptive task using a visual stimulus (a picture drawing of a living room).

For each audio file, the acoustic, lexical, rhythmic and syntactic linguistic features were extracted and analyzed. Due to the file's poor audio quality, 4 subjects belonging to the CG group were excluded from the analysis. The complete list of investigated features, the respective explanations and related references are reported in Supplementary Materials.

The statistical analysis was performed by comparing aMCI and mdMCI vs. CG by the non-parametric Kruskal-Wallis test, and eD vs. CG by the non-parametric Mann-Whitney *U*-test. Results are presented in Table 3, for 4 groups of features referred to the lexical (A), rhythmic (B), acoustic (C), and syntactic (D) features of the speech. The significant *p*-value is indicated for the corresponding feature and the corresponding speech task (figure, working day, dream).

**Table 3:** The table reports the results of the spontaneous speech analysis.

**(A) LEXICAL FEATURES**													
**Feature**	**Code**	**Figure**				**Working day**				**Dream**			
		**Kruskal-Wallis**	**Multiple comparison**		**Mann-Whitney**	**Kruskal-Wallis**	**Multiple comparison**		**Mann-Whitney**	**Kruskal-Wallis**	**Multiple comparison**		**Mann-Whitney**
			***aMCI***	***mdMCI***	***eD***		***aMCI***	***mdMCI***	***eD***		***aMCI***	***mdMCI***	***eD***
Content density	LEX_ContDens	0.0007	^**^	^**^	*U* = 98.5 *p* < 0.0001	0.711				0.9741			*U* = 102.5 *p* < 0.0001
Part-of-speech rate: Adj	LEX_PoS_ADJ	0.0086	^**^			0.0124	^*^			0.5366			*U* = 178 *P* = 0.0030
Part-of-speech rate: Adv	LEX_PoS_ADV	0.0968				0.4451			*U* = 193.5 *P* = 0.0072	0.2955			
Part-of-speech rate: art	LEX_PoS_ART	0.4329				0.9572			*U* = 185.5 *P* = 0.0047	0.7976			
Part-of-speech rate: conj	LEX_PoS_CONJ	0.5617				0.1266				0.0290	^*^		*U* = 225 *P* = 0.0331
Part-of-speech rate: interj	LEX_PoS_INTERJ	0.7401			*U* = 193 *P* = 0.0257	0.2145			*U* = 229.5 *P* = 0.0357	0.8830			
Part-of-speech rate: noun	LEX_PoS_NOUN	0.1714				0.1274			*U* = 130 *p* < 0.0001	0.2230			*U* = 223.5 *P* = 0.0310
Part-of-speech rate: num	LEX_PoS_NUM	0.4855				0.2719				0.9803			
Part-of-speech rate: phras	LEX_PoS_PHRAS	0.2351				0.2214				0.0403			*U* = 227 *P* = 0.0213
Part-of-speech rate: predet	LEX_PoS_PREDET	0.9299				0.5257			*U* = 219 *P* = 0.0122	0.7294			
Part-of-speech rate: prep	LEX_PoS_PREP	0.5495			*U* = 143 *P* = 0.0021	0.2078				0.2338			*U* = 212.5 *P* = 0.0187
Part-of-speech rate: pron	LEX_PoS_PRON	0.4161			*U* = 165.5 *P* = 0.0086	0.1433			*U* = 194.5 *P* = 0.0076	0.4150			
Part-of-speech rate: Vrb	LEX_PoS_VERB	0.1434				0.1103				0.0821			
Reference rate to reality	LEX_RefRReal	0.1089				0.0258		^*^	*U* = 174 *P* = 0.0024	0.3954			
Personal Deixis rate	LEX_PDEIXIS	0.7031			*U* = 189 *P* = 0.0217	0.1610			*U* = 182.5 *P* = 0.0039	0.5622			
Spatial Deixis rate	LEX_SDEIXIS	0.5189				0.5346				0.1813			
Temporal Deixis rate	LEX_TDEIXIS	0.7165				0.5619				0.1717			
Action verbs rate	LEX_ACTVRB	0.4493			*U* = 150.5 *P* = 0.0035	0.6362			*U* = 229 *P* = 0.0394	0.3406			
Propositional idea density	LEX_IDEAD	0.0125		^**^		0.8162				0.3547			*U* = 93.5 *P* < 0.0001
Frequency-of-use tagging	LEX_DM_F	0.2147				0.9209				0.3132			
Lexical richness: type-token ratio	LEX_TTR	0.0617				0.4601				0.336			
Lexical richness: Brunét's index	LEX_BrunetW	0.0144		^*^		0.3496			*U* = 219 *P* = 0.0257	0.1255			*U* = 188 *P* = 0.0054
Lexical richness: Honoré's statistic	LEX_HonoreR	0.5666			*U* = 129 *P* = 0.0008	0.8246				0.7209			
Mean number of words in utterances	LEX_NW	0.0071		^**^	*U* = 192.5 *P* = 0.0352	0.2203			*U* = 176 *P* = 0.0027	0.0670			
**(B) RHYTHMIC FEATURES**													
Percent. of vocalic intervals	RHY_%V	0.1749				0.7722				0.4195			
Std. deviation of vocalic interval durations	RHY_DeltaV	0.0709				0.2127				0.2484			
Std. deviation of consonantal interval durations	RHY_DeltaC	0.3722				0.8292				0.0011	^**^	^**^	
Pairwise variability index, raw	RHY_VnPVI	0.8350				0.5988				0.0314	^*^		
Pairwise variability index, normalized	RHY_CrPVI	0.4644				0.6684				0.0152	^*^		
Variation coefficient for ΔV	RHY_VarcoV	0.0079	^*^			0.2779				0.3225			
Variation coefficient for ΔC	RHY_VarcoC	0.2960				0.9642				0.0146	^*^		
**(C) ACOUSTIC FEATURES**													
Silence segments duration	SPE_SILMEAN	0.0028	^*^	^**^	*U* = 30 *P* = 0.0009	<0.0001	^**^	^***^	*U* = 73 *P* < 0.0001	0.0837			*U* = 147 *P* = 0.0004
Speech segments duration	SPE_SPEMEAN	0.0012	^*^	^**^	*U* = 85 *P* < 0.0001	0.0016	^*^	^**^	*U* = 113 *P* < 0.0001	0.0001	^**^	^***^	*U* = 115 *P* < 0.0001
Temporal regularity of voiced segment	SPE_TRVSD	0.7356				0.4391				0.4000			
Verbal rate	SPE_VR	0.0944			*U* = 99 *P* < 0.0001	0.0548			*U* = 150 *P* = 0.0005	0.0597			*U* = 109 *P* < 0.0001
Transformed phonation rate	SPE_TPR	0.0008	^*^	^**^	*U* = 105 *P* = 0.0001	0.0002	^**^	^**^	*U* = 91 *P* < 0.0001	0.0003	^**^	^**^	*U* = 98 *P* < 0.0001
Standardized phonation time	SPE_SPT	0.2824				0.1591				0.0840			
Standardized pause rate	SPE_SPR	0.0134		^*^	*U* = 96 *P* < 0.0001	0.0037	^*^	^*^	*U* = 109 *P* < 0.0001	0.0002	^**^	^**^	*U* = 69 *P* < 0.0001
Root mean square energy	SPE_RMSEM	0.2812				0.2035				0.2089			
Pitch	SPE_PITCHM	0.4208				0.3924				0.3847			
Spectral centroid	SPE_SPCENTRM	0.0162		^*^	*U* = 183 *P* = 0.0041	0.1505				0.1047			
Higuchi fractal dimension	SPE_HFractDM	0.0022		^***^		0.0022		^**^		0.0046		^**^	
**(D) SYNTACTIC FEATURES**													
Utterance length	SYN_SLENM	0.0596			*U* = 86 *P* < 0.0001	<0.0001	^*^	****	*U* = 85.5 *P* < 0.0001	0.0012	^**^	^*^	*U* = 110.5 *P* < 0.0001
Number of dependent elements linked to the noun	SYN_NPLENM	0.6967			*U* = 168 *P* = 0.0100	0.3866			*U* = 192.5 *P* = 0.0068	0.3754			
Global dependency distance	SYN_GRAPHDISTM	0.0275	^*^		*U* = 137 *P* = 0.0014	0.0106		^**^	*U* = 130 *P* = 0.0001	0.0010	^*^	^**^	*U* = 142 *P* = 0.0003
Syntactic embeddedness: maximum depth of the structure	SYN_MAXDEPTHM	0.1168			*U* = 122 *P* = 0.0005	0.0002	^*^	^***^	*U* = 76.50 *P* < 0.0001	0.0191	^*^		*U* = 134.5 *P* = 0.0002
Syntactic complexity	SYN_ISynCompl	0.3602				0.8648			*U* = 219.5 *P* = 0.0259	0.6019			*U* = 141.5 *P* = 0.0003

See Table in Supplementary Materials for abbreviations and references. For each group of features (lexical, rhythmic, acoustic, syntactic), the statistical analysis was performed by comparing aMCI and mdMCI vs. CG by the non-parametric Kruskal-Wallis tests and Dunn's multiple comparison, having the CG as “control group,” and by comparing CG vs. eD by the non-parametric Mann-Whitney U-test. The Kruskal-Wallis p-value, the significant p-values from the Dunn's test (aMCI and mdMCI) and Mann-Whitney U-test (eD) are reported in the table, where

* *p < 0.05*;

** *p < 0.01*;

*** *p < 0.001. aMCI, amnesic mild cognitive impairment; CG: control group; eD, early dementia; mdMCI, multiple domain mild cognitive impairment*.

The acoustic is the language category more altered in the PG. The acoustic parameters investigate temporal features, quantifying speech rate and pauses in the signal (e.g., silence segments duration, speech segments duration, verbal rate, transformed phonation rate, standardized phonation time, and standardized pause rate) as well as some spectral properties of the voice. Most of the considered acoustic features are mainly affected in the PGs compared to the CG group. Notably, these features seem to be able to distinguish, not only eD and mdMCI, but also aMCI from the CG.

On the contrary, the rhythmic parameters that quantify changes in speech rhythm and in utterance composition in terms of vowel and consonant alternation, are poorly modified during the cognitive decline, with the notable exception of the dream description task in both aMCI and mdMCI.

Lexical parameters, that attempt to gauge lexical knowledge and retrieval in spontaneous speech, are substantially altered in eD; among these features content density (i.e., the ratio of open-class words to closed-class words) is consistently reduced, especially for the picture description task, in both a- and mdMCI. In general, some features (namely LEX_ContDens, LEX_PoS_ADJ and the lexical richness parameters), indicating a global impoverishment of the speech production from the lexical point of view, resulted significant in distinguishing PG from CG. Reducing the amount of content words as well as using less modifiers (e.g., adjectives), results in poorer productions, even if the sentences are still correct from a formal point of view.

The syntactic features are designed to measure the degree of utterance structural complexity, and most of the investigated parameters are altered in the picture task in early AD subjects. Moreover, some syntactic features such as utterance length are altered not only in eD, but also in aMCI and mdMCI, using the “working day” and “dream” as speech tasks, which require the construction of a complete and structured narration, showing a general simplification. As a matter of fact, syntactic structures produced by PG, despite being grammatically correct and coherent, contains less complex relations among phrases and fewer embedded structures. Notably, narrative tasks, involving more than merely the naming of persons and objects as in the figure task, typically realized by both CG and PG in the form of coordinative structure or simple lists, are sensitive, with regard to syntactic features, in discriminating also aMCI.

## Discussion

The early recognition of cognitive decline is widely shared goal in the aging global population, and the focus is rapidly moving from defined clinical entities, such as MCI, to pre-clinical or asymptomatically stages in a general and still poorly defined frame addressed as “cognitive frailty” (Calzà et al., 2015). Specifically, early recognition helps to diagnose early dementia; identify dementia in at-risk individuals; design preventive clinical trials; identify reversible cognitive deficit in systemic diseases–metabolic, renal, cardiovascular, etc. – in depression or inappropriate pharmacological regimens; for secondary and tertiary prevention; and to define more appropriate health and social policies (Sugimoto et al., 2018; Vella Azzopardi et al., 2018).

Language has a central role among the cognitive domains that may reveal early signs of decline, becoming an established topic of research and clinical monitoring of AD progression (reviewed by Bucks et al., 2000; Kempler and Goral, 2008; Taler and Phillips, 2008; Shafto and Tyler, 2014; Szatloczki et al., 2015). Extensive literature on the use of traditional tests for the language assessment, especially with lexical and semantic access tasks, provides evidence that the lexico-semantic system is already affected in the initial stages of the disease, and patients have difficulties in tasks such as picture naming (Jacobson et al., 2002) and phonemic and semantic verbal fluency (Marczinski and Kertesz, 2006; Rascovsky et al., 2007). On the contrary, the phonological, morphological and syntactic systems are believed to be relatively preserved in the initial stages of the disease, as indicated by such tasks involving reading letters and words (Stilwell et al., 2016).

Studies dedicated to evaluate early language signs in prodromal or preclinical stages, such as MCI (Taler and Phillips, 2008; Drummond et al., 2015; Szatloczki et al., 2015; Hernández-Domínguez et al., 2018), reports inhomogeneous results. For example some authors described significant differences between controls and MCI in semantic fluency and naming tests (Ostberg et al., 2005; Duong et al., 2006; Radanovic et al., 2007; Cuetos et al., 2009; Joubert et al., 2010; Ahmed et al., 2013; Mueller et al., 2016), while others did not confirm differences in Boston Naming, semantic and phonemic verbal fluency (Bschor et al., 2001).

Moreover, the conventional neuropsychological language tests used in these studies fail to explore different levels of the cognitive network involved in complex linguistic activities (phonological, morphosyntactic, semantic-lexical, semantic-pragmatic). Thus, the spontaneous speech analysis is raising increasing interest in the neuropsychological research for the early detection of cognitive decline (Drummond et al., 2015; Aramaki et al., 2016; Pistono et al., 2016), also because of the high complexity of tasks that require not just lexical-semantic abilities, but also memory and executive functions. These novel approaches in language analysis may offer an opportunity to detect subclinical language changes, that may be present several years before the clinical phase of the disease and can be considered as one of the prodromal (or preclinical) manifestations of the disease.

The analysis at the discourse level is today possible by using the computational tools of NLP that allow the automatic detection of acoustic, lexical, semantic, syntactic and pragmatic parameters (Roark et al., 2011; Satt et al., 2013; König et al., 2015), thus leading to, a quantitative description and analysis of speech elicited by visual stimuli (“please describe this picture”), or by episodic memory (“please describe your last dream”). This approach has been also applied to the DementiaBank corpus, including narrative samples from 167 patients with “possible” or “probable” AD. By using two machine-learning classifiers, four factors distinguished AD vs. control narrative samples: semantic impairment, acoustic abnormality, syntactic impairment, and information impairment (Fraser et al., 2016). Other studies in small cohorts of AD patients (mild, moderate, and severe) have indicated alterations in articulation rate, speech tempo, hesitation ratio, and rate of grammatical errors (Hoffmann et al., 2010); and in acoustic measurements, such as pitch level, pitch modulation, and speaking rate (Horley et al., 2010). However, it should be noted that the potentiality of spontaneous speech analysis is poor in AD patients, even at early stages of the disease, due to the already severe alteration of the language performance.

Thus, increasing interest is directed toward subjects with subjective cognitive impairment or subjective memory complaints (Cuetos et al., 2009), a condition that could be a preclinical phase of the MCI condition (Jessen et al., 2014; Eichler et al., 2015; Mendonça et al., 2016), and MCI. In this proof-of-concept study we used the NLP tools for the analysis of the spontaneous discourse in early cognitive decline (aMCI and mdMCI) and in early Alzheimer disease (eD), included in the study as “positive control.” According to other studies, MCI group's results could represent an intermediate stage between CG and eD (Drummond et al., 2015). However, we demonstrated that aspects of the language not considered in conventional neuropsychological tests are deeply affected in MCI compared to CG. In particular, the acoustic features of language–e.g., pause duration, speech segment duration, and phonation rate-conveying linguistic and paralinguistic information such as illocution, modality, emphasis, attitude, and emotion (Finegan, 2011) seem to be sensitive markers of early cognitive decline, also distinguishing amnestic from multiple domain MCI. Notably, pause alterations during autobiographic discourse collected by the EPITOUL ecological task (exploring the episodic memory test) has been also described in MCI by others (Pistono et al., 2016). Consistent with previous scientific literature, the deterioration of verbal fluency, lexical retrieval process and discourse planning may result in longer hesitations, increased pauses and lower phonation rate. These acoustic features may discriminate between control groups and aMCI (König et al., 2015).

On the contrary, the speech rhythm seems to be rather preserved in the PGs included in our study, while in a study including eAD patients (MMSE > 24), a high variability of syllabic interval was reported (Martínez-Sánchez et al., 2017).

A number of studies have already demonstrated that lexical-semantic system is often impaired in MCI and dementia: our results confirm the finding, showing that patient's linguistic productions are semantically impoverished. Moreover, even though the correctness of grammatical form is generally preserved, syntax shows to be overall simplified.

Our study provides strong evidence to the emerging, but still puzzling literature, supporting spontaneous speech analysis as a potential tool for early detection of cognitive decline. The need to make these evaluation tools applicable on a large scale and at low cost, has prompted researchers to devise automated forms of analysis of collected samples of speech, recorded and manually transcribed according to appropriate coding systems, by software that can detect a series of acoustic and lexical variables, with detection of acoustic, lexical, semantic, syntactic, and pragmatic parameters (Thomas et al., 2005; Roark et al., 2011; Pakhomov et al., 2012; Satt et al., 2013). In these studies, speech was assessed through the collection of linguistic production samples obtained using various types of tasks.

An additional contribution from this study derived from the use of three different speech tasks. In spite of the fact that the description of a complex picture is the most widely used task (Goodglass et al., 2000; Bschor et al., 2001; Cuetos et al., 2009; König et al., 2015), we observed that the description of a “working day” and “the last dream” seem to be more sensitive tasks, probably because require memory recall and a more structured narration. Some other researchers have investigated different aspects of language as communication abilities (Toledo et al., 2018), reading comprehension (Hudon et al., 2006; Schmitter-Edgecombe and Creamer, 2010), the repetition of complex sentences (König et al., 2015; Lust et al., 2015) or the ability to recognize the grammatical correctness (Taler and Jarema, 2004). Other groups of researchers have applied the analysis of discourse on verbal productions recorded during the classic episodic memory tests as the Wechsler logical memory (Roark et al., 2007, 2011). The most ambitious studies have also used analytical tools applicable directly on the voice recordings of subjects (Meilán et al., 2014), showing a good correlation between the automated classification and that based on clinical and manual data processing (Roark et al., 2011; Satt et al., 2013; Hernández-Domínguez et al., 2018).

## Conclusion

Results from this proof-of-concept study suggest that computerized speech analysis identifies alterations in MCI for language features not explored by conventional diagnostic neuropsychological tests, also including language tests such as phonological and lexical fluency. Numerous acoustic features can distinguish between healthy controls and aMCI subjects, and lexical, rhythmic, and syntactic features may be also relevant, depending on the type of language task evaluated. While longitudinal studies *(in progress)* are necessary to confirm this hypothesis, we suggest that (i) speech analysis should be included as exploratory end-point in AD prevention studies; (ii) speech analysis coupled to imaging studies in cognitive decline could provide new information for language neuroanatomy; (iii) computerized speech analysis should be considered also for the development of novel tests for preclinical AD, thus contributing to the research priorities (prevention and identification of AD risk) identified by the WHO (Ministerial Conference on Global Action against Dementia, Shah et al., 2016).

## Author contributions

DB performed neuropsychological testing and speech recording, GG performed language analysis, RR worked out the linguistic planning and performed the critic review of the language data, EG performed the clinical study design, patient enrolment and neuropsychological test, FT performed language analysis and critical review of the language data, LC ideated and designed the study, analyzed, performed statistical analysis, interpreted the data and drafted the manuscript. All authors have contributed, read and approved the manuscript.

### Conflict of interest statement

The authors declare that the research was conducted in the absence of any commercial or financial relationships that could be construed as a potential conflict of interest. The reviewer GGE and handling Editor declared their shared affiliation.
