# Supplementary material for: Speech Analysis by Natural Language Processing Techniques: A Possible Tool for Very Early Detection of Cognitive Decline?
Source: Front Aging Neurosci. 2018 Nov 13;10:369. doi: 10.3389/fnagi.2018.00369 (PMC6243042; doi:10.3389/fnagi.2018.00369)
Supplement: Supplementary file 1 [file Table_1.pdf]

SUPPLEMENTARY MATERIALS
for the paper:

Daniela Beltrami, Gloria Gagliardi, Rema Rossini Favretti, Enrico Ghidoni, Fabio Tamburini and Laura Calzà (2018), "Speech analysis by Natural Language Processing techniques: a possible tool for very early detection of cognitive decline?" *Front. Aging Neurosci*. | doi: 10.3389/fnagi.2018.00369.

Description of the linguistic features investigated, relative label and range of results, as obtained in the study. Significant references are also included.

Acoustic features:

Feature	Description	Refs
**Silence segments duration**<br>**UM: sec.**	Silence segments of the signal identified using a voice activity detector (VAD). Mean, median and Std. Deviation were taken into account.	Satt *et al.,* 2013
**Speech segments duration**<br>**UM: sec.**	Speech segments of a signal identified using a voice activity detector (VAD). Mean, median and Std. Deviation were taken into account.	Satt *et al.,* 2013
**Temporal regularity of voiced segment**<br>**UM: quefrency**	The measure captures the temporal structure of the voiced segments, providing information on the rate of change in the different spectrum bands.<br>To calculate the temporal regularity of voiced segment durations, we used the sequence of the duration values, and calculated the real cepstrum of the sequence (i.e. the result of taking the inverse Fourier transform of the logarithm of the estimated spectrum of a signal).	Satt *et al.,* 2013
**Verbal Rate**<br>**U.M. : words/sec**	The number of words in the sample divided by the Total Locution Time (i.e. speech time including pauses).<br>#words/TLT	Singh *et al*., 2001; Roark *et al*., 2011
**Transformed Phonation Rate**<br>**UM: radians**	"The arcsine of the square root of the Phonation Rate."<br>$\arcsin(\sqrt{PR})$.<br>Where PR is the phonation rate<br>PR = TPT/TLT<br>TPT: total phonation time (i.e. speech time without pauses)<br>TLT: total locution time (i.e. speech time including pauses).<br><br>The arcsin transformation (or "angular transformation") provides a normally distributed measure within each participant group.	Singh *et al*., 2001; Roark *et al*., 2011
**Standardized Phonation Time**<br>**U.M. : words/sec**	The number of words in the sample divided by the total phonation time (i.e. speech time excluding pauses).<br>#words/TPT	Singh *et al*., 2001; Roark *et al*., 2011
**Standardized Pause Rate**<br>**U.M. : -**	The number of words in the sample divided by Pauses.<br>#words/#pauses	Singh *et al*., 2001; Roark *et al*., 2011
**Root Mean Square energy**<br>**U.M.: dB**	Physically, energy is a measure of "how much signal" exists at any one time, and it is used in continuous speech to detect voiced sounds, which have higher	López-de-Ipiña *et al.*, 2013

	intrinsic energy than unvoiced segments.<br>The energy of a signal is typically calculated by windowing the signal at a particular time, squaring the samples and taking the average. The square root of this result is the engineering quantity known as the root-mean square (RMS) value.<br>Mean and Std. Deviation of the measures were taken into account.	
**Pitch**<br>**UM: Hz**	Pitch is the main acoustic correlate of tone and intonation, and the perceptual correlate of frequency; as a matter of fact, it depends on the number of vibrations per second produced by the vocal cords.<br>Mean and Std. Deviation were taken into account.	López-de-Ipiña *et al.*, 2013
**Spectral Centroid**<br>**UM: Hz**	The measure captures the perceptual brightness of a sound. It is obtained by evaluating the "centre of gravity" of the spectrum using the Fourier transform's frequency and magnitude information.<br>Mean and Std. Deviation were taken into account.	López-de-Ipiña *et al.*, 2013
**Higuchi Fractal Dimension**<br>**UM: pure number**	The feature describes the complexity of the signal. The algorithm measures fractal dimension (i.e. self-similarity, namely identical/similar structures repeating over a pattern) of discrete time sequences directly from time series.<br>Mean and Std. Deviation were taken into account.	López-de-Ipiña *et al.*, 2013

Rhythmic features:

**Feature**	**Description**	**Refs**
**Percentage of vocalic intervals**<br>**UM: %**	The proportion of vocalic intervals within the utterance, that is, the sum of vocalic intervals divided by the total duration of the utterance.	Ramus *et al.*, 1999
**Std. deviation of vocalic and consonantal interval durations**<br>**UM: msec**	The standard deviation of the duration of vocalic and consonantal intervals within each utterance, noted as ΔV and ΔC.	Ramus *et al.*, 1999
**Pairwise Variability Index, raw and normalized**<br>**UM: msec**	This rhythm metric takes into account the temporal succession of the vocalic and consonantal intervals instead of joining all the values and calculating the standard deviation. It is based on a pairwise comparison of the durations of either two vocalic or consonantal intervals, therefore expressing the level of variability in consecutive measurements.<br><br>• Raw Pairwise Variability Index (*rPVI*):<br>$r\ PVI = \left[\sum_{k=1}^{m-1} \lvert d_k - d_{k+1} \rvert / (m-1) \right]$<br>where *m* is number of intervals, vocalic or intervocalic, in the text and *d* is the duration of the *k*th interval.<br><br>• Normalised Pairwise Variability Index (nPVI):<br>n<br>$PVI = 100 \times \left[\sum_{k=1}^{m-1} \left\lvert \frac{d_k - d_{k+1}}{(d_k + d_{k+1})/2} \right\rvert / (m-1) \right]$	Grabe & Low, 2002

**Variation coefficient for ΔV and ΔC**<br>**UM: pure number**	A variation coefficient ("varco") is a value describing relative variation. VarcoΔC is calculated as the percentage of the ΔC of the average duration of intervals (meanC); analogously, VarcoΔV is calculated as the percentage of the ΔV of the average duration of intervals (meanV).<br><br>VarcoΔC= ΔC*100/meanC<br>VarcoΔV= ΔV*100/meanV	Dellwo, 2006

Lexical Features

Feature	Description	Refs
**Content Density**<br>**U.M: pure number**	The ratio of open-class words to closed-class words. The measure is calculated over Part of Speech tags, where open-class words are nouns, verbs, adjectives, adverbs; the rest are considered closed-class words.<br><br>Content Density=OCW/ CCW	Roark *et al.*, 2011
**Part-of-Speech rate**<br>**U.M: %**	This class of features investigates the average rate of occurrence for each part-of-speech (PoS) category: Adjectives, Adverbs, Articles, Conjunctions, Interjections, Nouns, Numerals, Prepositions, Pronouns, Verbs.<br><br>e.g.: #Adjectives/#words	Holmes & Singh, 1996;<br>Bucks *et al.*, 2000
**Reference Rate to Reality**<br>**U.M: pure number**	The ratio of the total number of nouns to the total number of verbs.<br>RefRReal = #Nouns/#Verbs	Vigorelli, 2004
**Personal, Spatial and Temporal Deixis rate**<br>**U.M: %**	The feature probes the rate of deictic expressions in the spoken text (i.e. linguistic elements that point to the time, place, or situation in which a speaker is speaking; in other words, their denotational meaning varies depending on extralinguistic context). The main types of deixis are:<br>• Person deixis (e.g. *I, you, we, me, mine, yours*…)<br>• Place deixis (e.g. *here, there, this, that…*)<br>• Time deixis (e.g. *now, today, tomorrow, soon*…)<br><br>e.g.: #PersonDeixis/#words	March *et al.*, 2006;<br>Cantos-Gomez *et al.*, 2009
**Relative pronouns and negative adverbs rate**<br>**U.M: %**	The rate of Relative Pronouns (e.g. *who, whose*…) and Negative Adverbs (e.g. *not, neither…*) in the spoken text.	
**Lexical Richness: Type-Token Ratio, W - Brunet's Index and R - Honoré's Statistic**<br>**U.M.: pure number**	This class of measures quantifies the richness of vocabulary/lexical diversity.<br><br>**Type-Tokes Ratio**: the ratio of the number of different words (vocabulary - V) to the total text length.<br>TTR is dependent on the text size: it is bigger when texts are small and decreases as the texts get larger.<br><br>**W - Brunet's Index:** it quantifies lexical richness without being sensitive to text length. It is calculated according to the following equation:<br>$W = {}_N V^{(-.165)}$<br>where N is the total text length and V is the total	Holmes & Singh, 1996;<br>Brunet, 1978;<br>Honoré, 1979;

	vocabulary used by the participant.<br>This measure generally varies between 10 and 20. The lower the value, the richer the speech.<br><br>**R - Honoré's Statistic:** calculates lexical richness by highlighting the proportion of words that are used only once with reference to the total number of words in the text: the larger the number of words used by a speaker that occur only once (hapax legomena), the richer the lexicon.<br>$R = 100 \log N / (1 - V1/V)$<br>where V1 is the words spoken only once, V is the total vocabulary used and N is the total text length.<br>High value of R suggests a rich vocabulary used by the speaker.	
**Action Verbs rate**<br>**U.M: %**	The metric probes the rate of action verbs (i.e. verbs referring to physical action, like *to put, to run, to eat*) in the spoken text.	
**Frequency-of-use tagging**<br>**U.M: pure number**	Mean frequency-of-use weight among words extracted from the De Mauro's frequency list.	De Mauro, 2000;
**Propositional Idea Density**<br>**U.M: pure number**	Idea density is the number of expressed propositions (i.e. distinct facts or notions contained in a text) divided by the number of words. It is a measure of the extent to which the speaker is making assertions (or asking questions) rather than just referring to entities.<br>In this feature, propositions correspond to verbs, adjectives, adverbs, prepositions, and conjunctions. Nouns are not considered to be propositions, as the main verb and all its arguments count as one proposition.	Snowdon *et al.*, 1996; Roark *et al.*, 2011
**Mean Number of words in utterances**<br>**UM: pure number**	Mean number of words in the speech utterances.	

Syntactic features.

**Feature**	**Description**	**Refs**
**Number of dependent elements linked to the noun**<br>**U.M: words**	The feature explores Noun Phrase complexity, counting the number of dependent elements linked to the head (e.g. Adjectives, Relative clauses…).<br>Mean and Std. Deviation were taken into account.	
**Global Dependency Distance**<br>**U.M: pure number**	Given the memory overhead of long distance dependencies, the feature quantifies the difficulty in syntactic processing.<br>Mean and Std. Deviation were taken into account.	Roark *et al.*, 2007; 2011
**Syntactic complexity**<br>**U.M: pure number**	Syntactic complexity is established by counting the linguistic tokens that can be considered to telltale signs of increased grammatical subordinateness and embeddedness, such as:<br>1. subordinating conjunctions (e.g. *because, since, as, when, that*, etc.);<br>2. WH- pronouns (e.g. *who, whose, whom, which*);<br>3. verb forms, both finite and non-finite;<br>4. noun phrases.<br>Because subordinators and WH-pronouns are the most straightforward indicators of increased embeddedness (and thus of high complexity), these	Szmrecsanyi, 2004

	features are weighted more heavily than verb forms and noun phrases.<br>(2*CONJ+2*PRON+NOUNS+VERBS)/#word	
**Syntactic embeddedness: maximum depth of the structure**<br>**U.M: pure number**	Syntactic complexity is also assessed by evaluating the "embeddedness", i.e. the maximum "depth" of the structure.<br>Mean and Std. Deviation were taken into account.	
**Utterance length**<br>**U.M: word/utterance**	Mean Length of utterance corresponds to the average number of words for utterance. It is calculated by counting the number of words in each utterance divided by the total number of utterances.<br>Mean and Std. Deviation were taken into account.	

ADDITIONAL REFERENCES

Brunet, E. (1978) *Le Vocabulaire de Jean Giraudoux. Structure et Evolution*. Geneve: Slatkine.

Cantos-Gòmez, P. (2009) Featuring linguistic decline in Alzheimer's disease: A corpus-based approach. In Mahlberg, M., Gonzàlez Dìaz, V., Smith, C. (eds.), *Proc. of the Corpus Linguistics Conference 2009* (CL2009), pp1-12.

De Mauro, T. (2000). *Il dizionario della lingua italiana*. Paravia.

Delwo, V. (2006) Rhythm and speech rate: a variation coefficient for deltaC. In Karnowski, P., Szigeti, I. (eds.), *Language and Language-Processing*, Frankfurt am Main: Peter Lang, 231-241.

Grabe, E., and Low, E. L. (2002) Durational variability in speech and the rhythm class hypothesis. In: Gussenhoven, C., Warner, N. (eds), *Papers in Laboratory Phonology 7*, Berlino: Mouton de Gruyter, 515-546.

Holmes, D. I., and Singh, S. (1996) A stylometric analysis of conversational speech of aphasic patients. *Literary and Linguistic Computing*, 11(3): 133–140.

Honoré, A. (1979) Some simple measures of richness of vocabulary. *Association of Literary and Linguistic Computing Bulletin*, 7: 172–177.

López-de Ipiña, K., Alonso, J.B., Travieso, C.M., Solé-Casals, J., Egiraun, H., Faundez-Zanuy, M., et al. (2013) On the selection of non-invasive methods based on speech analysis oriented to automatic Alzheimer disease diagnosis. Sensors, 13:6730–6745.

March, E.G., Wales, R., Pattison, P. (2006). The uses of nouns and deixis in discourse production in Alzheimer's disease. In *Jour. of Neurolinguistics*, 19: 311–340.

Ramus, F., Nespor, M., Mehler, J. (1999). Correlates of linguistic rhythm in the speech signal. *Cognition* 73:265-292.

Singh, S., Bucks, R. S., and Cuerden, J. M. (2001). An evaluation of an objective Q17 technique for analysing temporal variables in DAT spontaneous speech. *Aphasiology* 15, 571–583. doi: 10.1080/02687040143000041.

Snowdon, D. A., Kemper, S. J., Mortimer, J. A., Greiner, L. H., Wekstein, D. R., Markesbery, W. R. (1996) Linguistic ability in early life and cognitive function and Alzheimer's disease in late life: Findings from the nun study. *JAMA*, 275:528–532.

Szmrecsanyi, B.M. (2004). On operationalizing syntactic complexity. In Purnelle, G., Fairon, C., Dister, A. (eds), *Proc. of the 7th International Conference on Textual Data Statistical Analysis*, Presses Universitaires de Louvain, 1031–1038.

Vigorelli, P. (ed.) (2004). *La conversazione possibile con il malato Alzheimer*, Franco Angeli.
